# Alginate Edible Films Containing Essential Oils: Characterization and Bioactive Potential

**DOI:** 10.3390/polym17091188

**Published:** 2025-04-27

**Authors:** Ana I. Lopes, Adma Melo, Tiago B. Afonso, Sara Silva, Lillian Barros, Freni K. Tavaria, Manuela Pintado

**Affiliations:** 1 CBQF—Centro de Biotecnologia e Química Fina—Laboratório Associado, Escola Superior de Biotecnologia, Universidade Católica Portuguesa, Rua Diogo Botelho 1327, 4169-005 Porto, Portugal; anlopes@ucp.pt (A.I.L.); tbafonso@ucp.pt (T.B.A.); snsilva@ucp.pt (S.S.); ftavaria@ucp.pt (F.K.T.); mpintado@ucp.pt (M.P.); 2 Centro de Investigação de Montanha (CIMO), Instituto Politécnico de Bragança, Campus de Santa Apolónia, 5300-253 Bragança, Portugal; lillian@ipb.pt; 3 Laboratório Associado para a Sustentabilidade e Tecnologia em Regiões de Montanha (SusTEC), Instituto Politécnico de Bragança, Campus de Santa Apolónia, 5300-253 Bragança, Portugal

**Keywords:** edible films, essential oils, antimicrobial activity, antioxidant activity, film’s characterization

## Abstract

Essential oils (EOs) are natural substances rich in phenolic compounds with notable antimicrobial and antioxidant properties. However, they present some limitations, such as low stability and bioavailability. Incorporating EOs into polymeric films offers a novel approach to overcome these challenges while enhancing their efficacy. In this study, we produced and thoroughly characterized alginate-based edible films incorporated with five different EOs—rosemary, eucalyptus, oregano, sage, and thyme. This is the first comprehensive investigation to include this diverse range of EOs in alginate films. Their antimicrobial and antioxidant activities were also evaluated. The results demonstrated that alginate films containing EOs exhibited significant bioactive properties. Notably, the film incorporated with oregano EO completely inhibited the growth of all tested bacteria and fungi and showed the highest antioxidant activity. Based on these findings, alginate films containing EOs present promising bioactive potential and could serve as biodegradable alternatives to conventional packaging materials, reducing environmental impact. However, further studies are necessary to assess their safety profile and confirm their viability as replacements for traditional food packaging. Future research should focus on evaluating cytotoxicity, genotoxicity, and the practical application of these films in food matrices.

## 1. Introduction

In recent years, plastic materials have been on the agenda. In fact, 8.3 billion tons of plastic are produced worldwide, with traditional food packaging accounting for 36.9% of plastic production, and 6.3 billion tons become waste, resulting in a serious environmental problem [1,2]. Additionally, the increasing demand for food products with higher quality and fewer synthetic additives and preservatives [3] has prompted the scientific community to intensify efforts to create new food packaging materials.

Edible films can be an alternative to traditional food packaging since they are renewable, non-toxic, biocompatible, and environmentally friendly. They act as a barrier to gases and protect food products from dehydration [3,4]. These formulations are made from biopolymers, such as polysaccharides, proteins, and lipids. Alginate is a biopolymer widely used in edible film formation due to its excellent gel-forming properties. It is generally recognized as a safe (GRAS) material with good biocompatibility, biodegradability, and low cost [5]. This polysaccharide is produced by brown algae from the Phaeophyceae class and by soil bacteria such as Azotobacter vinelandii and Pseudomonas aeruginosa [6,7].

Edible films can be incorporated with antioxidant and antimicrobial substances, such as essential oils (EOs), resulting in bioactive packaging materials. EOs are natural liquid mixtures of volatile molecules, such as terpenes, terpenoids, and phenolics [8,9]. They are synthesized by all plant organs (buds, flowers, leaves, stems, twigs, seeds, fruits, roots, wood, and bark) and stored in different structures, such as secretory cells, cavities, canals, epidermic cells, and glandular trichomes [9,10]. EOs are considered GRAS substances [11], but their strong flavor restricts their application as food preservatives [12].

The incorporation of EOs into polymeric films increases their stability, bioactivity, and antimicrobial potential [13]. While high concentrations of EOs are usually required to achieve desired bioactive effects, their application in edible films reduces the need for such amounts. This is because edible films act as controlled-release systems, allowing for the gradual release of EOs onto food surfaces [8,11]. Additionally, incorporating EOs into edible films helps mitigate their strong flavor [12].

The main objective of this study was to develop natural polymeric films based on alginate for potential food applications. The films were enriched with EOs of rosemary, eucalyptus, oregano, sage, and thyme—plants that are abundant in Portugal. These oils are particularly rich in oxygenated monoterpenes, including carvacrol in oregano, thymol in thyme, eucalyptol in both rosemary and eucalyptus, and camphor in sage. These compounds are primarily responsible for the notable antimicrobial and antioxidant properties of these EOs [14]. The films were characterized using Fourier Transform Infrared (FTIR) spectroscopy to evaluate structural modifications and assess the compatibility between the alginate matrix and the incorporated EOs. Additionally, their moisture content, solubility, wettability, and water vapor permeability were analyzed. Antimicrobial activity was assessed using the viable cell count method for bacteria and by evaluating fungal growth on the film surface. Antioxidant activity was determined using the kinetic ABTS radical scavenging assay. These three approaches are not commonly used in similar studies, highlighting the distinctiveness of the methodology applied.

## 2. Materials and Methods

### 2.1. Materials

Sodium alginate and sodium tripolyphosphate (TPP; 85%) were obtained from Sigma-Aldrich Chemie Gmbh. (St. Louis, MO, USA). Glycerol (99.5%) was purchased from Merck (Darmstadt, Germany). Müller–Hinton (MH) broth and agar culture media were acquired from BIOKAR Diagnostics (Allonne, France), and Sabouraud dextrose agar (SDA) was obtained from Laboratorios Conda (Madrid, Spain). Rosemary, eucalyptus, oregano, sage, and thyme EOs were provided by Instituto Politécnico de Bragança (Bragança, Portugal). Their chemical characterization is reported in Spréa et al. [14].

### 2.2. Preparation of Alginate Films Incorporated with EOs

Rosemary, eucalyptus, oregano, sage, and thyme EOs were selected for film production. Alginate films were produced using the traditional casting method [15]. A film-forming solution was prepared by dissolving sodium alginate (3% *w*/*v*) in hot distilled water (approximately 70 °C), followed by the addition of glycerol (2.5% *v*/*v*, based on the total volume of the solution) as a plasticizer. The solution was stirred continuously at room temperature (~22–25 °C) for 2 h to ensure complete dissolution.

After stirring, a 1% (*w*/*v*) aqueous solution of sodium tripolyphosphate (TPP), used as a cross-linking agent, was added dropwise to the alginate–glycerol mixture under constant stirring. The amount of TPP solution added was calculated to maintain a final concentration of 1% (*w*/*v*) TPP relative to the total film-forming solution volume.

To produce films containing EOs, selected oils were incorporated at 3% (*v*/*v*), calculated based on the total volume of the film-forming solution. The oils were added after the TPP incorporation to minimize loss of volatile components, and the mixture was stirred until the oil was fully emulsified.

Aliquots of 20 mL of each final solution were poured into 10 cm diameter plastic Petri dishes and left to dry at room temperature for four days. The resulting dried films had an average weight of approximately 1.316 ± 0.03 g. Based on estimated retention of each component during the drying process (100% retention for sodium alginate and TPP, ~80% for glycerol, and ~30% for essential oil), the final dry film composition was approximately as follows: sodium alginate (40.9% *w*/*w*), glycerol (34.4% *w*/*w*), TPP (13.6% *w*/*w*), and EO (11.0% *w*/*w*).

### 2.3. Characterization of the Films

**Figure 1:**
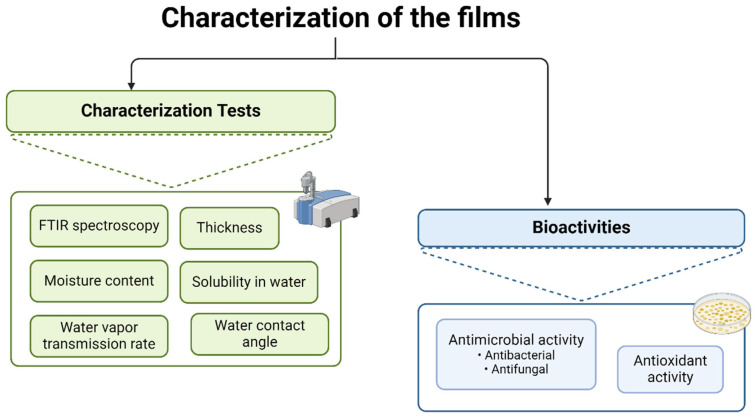
A schematic representation of the experimental procedure used for film characterization.

All films, with and without EOs, were characterized according to the scheme outlined in Figure 1. The film without EO served as the control in all characterization tests and will be referred to as “Control” from this point forward.

#### 2.3.1. FTIR Spectroscopy

The spectra of the films containing EOs were obtained with an FTIR spectrometer (PerkinElmer Precisely Spectrum 100, Waltham, MA, USA) supplied with an attenuated total reflectance (ATR) accessory (PIKE MIRacle™, PIKE Technologies, Fitchburg, WI, USA) with a diamond crystal plate. The spectra were acquired with 32 scans and 4 cm^−1^ resolution in the 4000–500 cm^−1^ region. An alginate film without EOs was also analyzed and used as a control to examine the structural changes in the alginate films resulting from the addition of oils.

#### 2.3.2. Film Thickness

The thickness of each film was determined through four separate measurements, taken randomly at various locations on each film, using a micrometer Model m120 (from Adamel Lhomargy, Roissy en Brie, France).

#### 2.3.3. Moisture Content and Solubility in Water

The moisture and solubility percentages of the films were determined according to Pereira et al. [16], with some modifications.

First, all films were weighed using an analytical scale (ACJ 120-4M, Kern & Sohn, Balingen, Germany). Then, they were put on previously dried and weighed glass Petri dishes and left to dry in an oven at 105 °C for 24 h until a constant weight was achieved. The moisture content, expressed as a percentage of weight loss after drying, was determined and reported on a dry basis, following Equation (1):

Moisture (%) = (M1 − (M3 − M2)) × 100(1)

where M1 is the initial mass of the film (before drying), M2 is the mass of the Petri dish, and M3 is the mass of the Petri dish with the film after drying.

To determine the solubility of the films in water, the previously dried samples were soaked in 35 mL of distilled water at 25 °C for 5 min. Afterwards, the films were filtered using Whatman No. 1 filter paper and dried at 105 °C for an additional 24 h. The solubility was calculated using Equation (2):

Solubility (%) = ((M4 − M5)/M4) × 100(2)

where M4 is the mass of the film after drying for 24 h at 105 °C, and M5 is the mass of the insoluble part of the film. Both analyses were conducted in triplicate for each film.

#### 2.3.4. Water Vapor Transmission Rate

The water vapor permeability rate (WVPR) was determined gravimetrically using the method described by ASTM [17] with some modifications.

For this test, 125 mL Erlenmeyers were filled with 50 mL of distilled water. Then, the films were fixed to the opening of the Erlenmeyers, ensuring that all the moisture/water vapor migration occurs through the film exclusively. All Erlenmeyer–water–film systems were weighed on an analytical scale (Mi) and stored in a desiccator containing silica gel at 25 °C. All systems were weighed daily (Mf) for five days. The water vapor transmission rate was calculated using Equation (3):

WVP = (M_i_ − M_f_)t × A (3)

where M_i_ is the initial mass of the system, M_f_ is the final mass of the system, t is the time (in days), and A is the area of the Erlenmeyer’s opening. Each film was tested in triplicate.

#### 2.3.5. Water Contact Angle (WCA) Measurements

The WCAs of the films with EOs and of the control film were determined with a tensiometer (Attension Theta, Biolin Scientific, Gothenburg, Sweden) by the sessile drop technique (Laplace–Young method). Each film was tested in triplicate by dispersing a 3 µL droplet on the film surface and measuring the angles formed between the baseline and the tangent lines to the water droplet surfaces. The mean WCA for each film was calculated by determining the means of the values obtained after 5 s of stabilization of the water droplets.

### 2.4. Bioactivities

#### 2.4.1. Antibacterial Activity

The antibacterial activity of the films with EOs and the film without an antimicrobial agent was determined against the following bacteria: *Bacillus cereus* (*B. cereus*), *Listeria monocytogenes* (*L. monocytogenes*), *Staphylococcus aureus* (*S. aureus*), *Escherichia coli* (*E. coli*), *Pseudomonas aeruginosa* (*P. aeruginosa*), *Salmonella enterica* serovar enteritidis (*S. enterica* serovar enteritidis) and *Yersinia enterocolitica* (*Y. enterocolitica*).

A viable cell test, as described in a previous study [15], was performed because this method allows for a more realistic determination of the antibacterial activity of the films. In this test, the films were left in contact with the bacterial inoculum (10^6^ CFU/mL) for different time points (0, 2, 4, 6, 8, 16, 24 h) at 37 °C. After the incubation, the films were dissolved in peptone water; 4 ten-fold dilutions were then undertaken in the same solution. Then, 20 µL aliquots of each dilution were pipetted on Mueller–Hinton agar plates, incubated at 37 °C for 24 h, and the colonies were counted. The results were expressed as log CFU/g.

#### 2.4.2. Antifungal Activity

The antifungal activity was determined against *Aspergillus niger* (*A. niger*), *Penicillium expansum* (*P. expansum*), *Fusarium verticillioides* (*F. verticilllioides*), and *Cladosporium* sp. The method applied by the authors in a previous study [15] evaluated the ability of the fungi to grow on the film’s surface. In this test, film discs were placed in the center of SDA plates and inoculated with a spore suspension containing 10^5^ spores/mL. Then, the plates were incubated at 25 °C for seven days, and the diameter of the fungal colony was measured. The percentage of inhibition was calculated by comparing the reduction in fungal growth in films with EOs to the growth in the control film, following Equation (4):

Fungal growth inhibition (%) = [(D_c_ − D_f_)/D_c_] × 100 (4)

where D_c_ is the diameter of the fungal colony in the control film, and D_f_ is the diameter of the fungal colony in the film with EO.

#### 2.4.3. Antioxidant Activity

The antioxidant activity of the films with EOs and the control film was evaluated using the 2,2′-azino-bis (3-ethylbenzothiazoline-6-sulfonic acid) (ABTS) scavenging assay. This technique measures the antioxidant potential by monitoring the inhibition of the cationic radical ABTS+·, with the absorbance recorded spectroscopically at 734 nm.

Before the test, the ABTS radical cation was prepared by mixing 7.0 mM ABTS with 2.44 mM potassium persulfate (1:1, *v*/*v*) and allowing the mixture to react in the dark at room temperature for 16 h. The ABTS solution was then diluted with methanol to achieve an absorbance of 1.0 at 734 nm.

For the assay, fixed volumes of each film-forming solution were pipetted into wells of a 24-well microplate and dried at room temperature until small films formed. Next, 2 mL of the ABTS+· radical solution was added to the films. The reduction in absorbance was measured kinetically over 12 min at 50 s intervals using a Synergy H1 plate reader (Winooski, VT, USA). Each film was tested five times.

### 2.5. Statistical Analysis

The results were presented as mean ± standard deviation (SD). Statistical analysis was conducted using GraphPad Prism 8.0.1 (GraphPad^®^ Software, San Diego, CA, USA). A one-way analysis of variance (ANOVA) was performed, followed by Tukey’s HSD post hoc test, after confirming homoscedasticity. A non-parametric test, followed by the Kruskal–Wallis test, was also used after confirming non-homoscedasticity.

## 3. Results

### 3.1. Alginate Films with EOs

EOs, due to their high content of phenolic compounds, exhibit strong antimicrobial and antioxidant activities, making them effective preservative agents [18]. However, the intense flavor and the necessity for elevated concentrations to attain desired effects constitute two drawbacks to their utilization [7]. The incorporation of EOs on polymeric films decreases their sensory impact and inhibits their diffusion into food products [7,11]. Additionally, the application of EOs in films ensures their gradual release onto food surfaces and within packages headspaces, requiring smaller amounts of oil [7].

**Figure 2:**
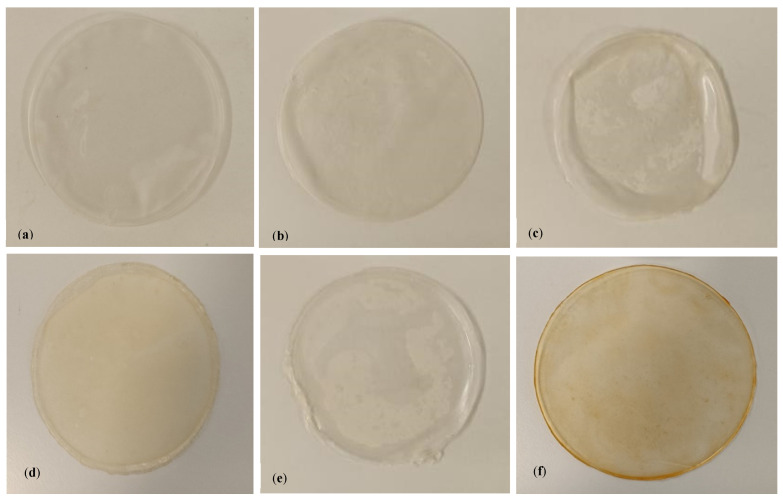
Photographs of the alginate films. (**a**) Alginate film without EO; (**b**) alginate film with rosemary EO; (**c**) alginate film with eucalyptus EO; (**d**) alginate film with oregano EO; (**e**) alginate film with sage EO; (**f**) alginate film with thyme EO.

In this study, rosemary, eucalyptus, oregano, sage, and thyme EOs were incorporated into alginate films (Figure 2). The concentration of oil on the films was 3% (*v*/*v*). An alginate film without EO was also produced and used as a control in the antimicrobial and antioxidant tests.

### 3.2. Characterization of the Films

#### 3.2.1. FTIR Spectroscopy Analysis

**Figure 3:**
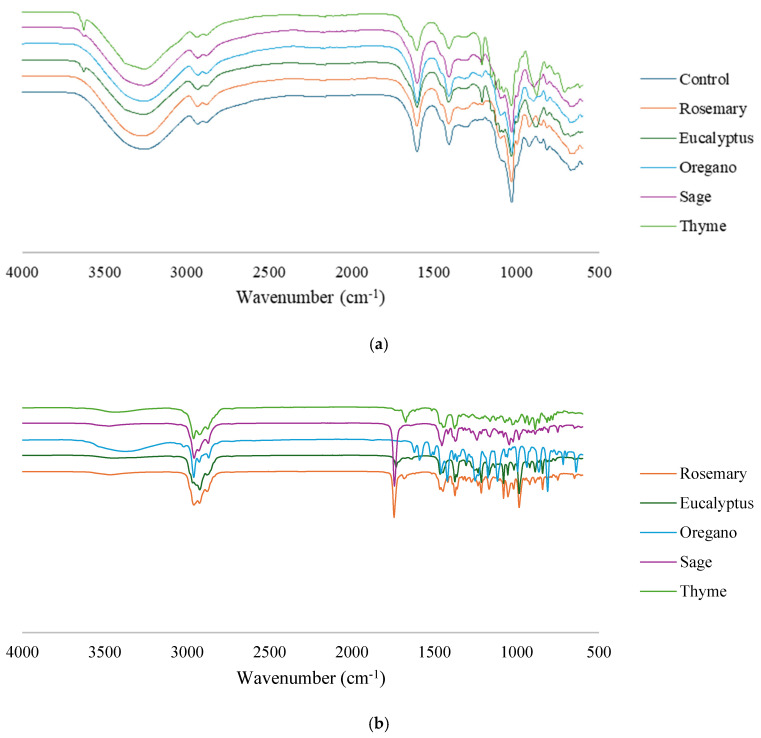
(**a**) FTIR spectra of the alginate and TPP film (control film) and the films incorporated with EOs; (**b**) FTIR spectra of the pure EOs.

The infrared spectra of the control film and the films incorporated with EOs (Figure 3) were used to investigate the intermolecular interactions between the films’ components and assess whether the addition of an EO to the film altered its structure. Additionally, the FTIR spectra of the pure EOs (Figure 3b) were examined to help identify specific functional groups responsible for any spectral changes observed in the films, thereby providing further insight into the interactions between the oils and the film structure.

The FTIR spectra of all films exhibited absorption peaks typical of the functional groups present in alginate. In fact, the peaks at 1600–1603 cm^−1^ (asymmetric stretching of COO- groups), 869 cm^−1^ (Cl-H of the α-L-guluronic residue), and 815 cm^−1^ (mannuronic acid residues/C–O–C symmetric stretching) indicate the presence of uronic acid [18,19]. Additionally, the presence of intense peaks at 2921–2930 cm^−1^ corresponds to the symmetric vibrations of alginate C-H groups [18,20,21], and the peaks at 1026–1031 cm^−1^ are indicative of glycosidic bonds in the polysaccharide chain [19,20]. Peaks at 924 cm^−1^ and 851 cm^−1^ indicate the O-H deformation and C-O symmetric stretching in glycerol, respectively [19]. In all spectra, it is also possible to observe a broad peak in the 3200–3600 cm^−1^ range that is consistent with the -OH stretching vibrations and hydrogen bonds among hydroxyl groups of water and emulsified oil droplets [13,20,22,23].

The FTIR analysis also showed that adding an EO to a film did not significantly alter its chemical composition since all spectra were very similar. An identical result was reported in other studies [13,18] and may indicate that (i) the chemical structure of the control film is stable and does not alter significantly when EOs are incorporated; (ii) EOs are completely incorporated into the alginate matrix of the film; and/or (iii) the concentration of EO in the film is quite low (when compared to other components) and therefore not sufficient to cause visible changes in the FTIR spectrum [18].

However, some subtle variations were observed in the spectra of the films with EOs, suggesting the presence and partial incorporation of EO-specific functional groups into the alginate matrix. The film with rosemary oil exhibited slightly more intense bands around 1740 cm^1^ and 1100 cm^1^, corresponding to C=O and C–O stretching from esters and terpenes such as rosmarinic acid [24]. Eucalyptus film showed minor increases near 1040–1215 cm^1^, consistent with the presence of cyclic ethers like 1,8-cineole [25]. Oregano and thyme films displayed increased intensity around 1500–1600 cm^−1^, corresponding to aromatic C=C stretching from phenolic compounds like carvacrol and thymol [26]. The sage film showed slight broadening and shifting in the O–H and C–O regions (~3400 cm^1^ and ~1100 cm^1^), likely due to monoterpenes and alcohols interacting with the alginate [27].

#### 3.2.2. Thickness, Moisture Content, and Solubility in Water

Edible films, to be effective food packaging materials, must have adequate resilience to external factors but also be elastic and resistant throughout the processes of packaging and storage [16].

Thickness is an essential characteristic of the films since it affects their mechanical properties [16]. The thickness of the alginate films with EOs and the film without oil were determined, and the results are presented in Table 1.

The film values of thickness ranged from 0.159 mm to 0.291 mm. Adding thyme EO into an alginate and TPP film decreased its thickness, although the difference was not significant (*p* > 0.05), as also observed for the rosemary-containing film. On the other hand, incorporating eucalyptus, oregano, and sage EOs into films significantly increased their thickness (*p* < 0.05). A similar result was reported by Bhatia et al. [22] for alginate/acacia gum films incorporated with cinnamon EO. The increase in the viscosity of film-forming solutions with EOs may explain the rise in thickness of the films [22].

Moisture is an important parameter of films’ characterization, as it can be an indication of their hydrophobicity [18]. The moisture content of the films with and without EOs varied from 29.54% to 37.79% (Table 1). The control film had the lowest moisture content, while films with EOs presented higher moisture percentages; this increase in moisture was significantly different (*p* < 0.05) for the films with eucalyptus, oregano, sage, and thyme. The addition of EOs into the films is the reason behind the different moisture values observed. These substances can change the hydrophilicity of the films, thus affecting their permeability to water [28].

Water solubility is an essential factor in the preparation of films for food applications. Usually, films with low solubility are required in order to improve water resistance and integrity of the packaging materials. However, for the encapsulation of foods, films with high solubility may be beneficial [7,16]. The control film (i.e., the film without EO) was the most soluble (solubility value of 95.10%). This behavior was probably a result of the hydrophilic nature of the alginate [19]. The solubility of the films with EOs ranged from 83.72% (film with oregano oil) to 94.29% (film with sage oil). The solubility of the films decreased in the films with EOs, probably because of the hydrophobic nature of the oils [7]. However, the differences in the solubility values between the control film and the films with EOs were not significant (*p* > 0.05). This is probably because the amount of oil added to the films was not high enough to significantly affect their hydrophobicity [13,19].

In summary, the incorporation of EOs into the films affected their physical properties, including thickness, moisture content, and water solubility. A positive correlation was observed between film thickness and moisture content, as seen in the eucalyptus and sage films, which exhibited both higher thickness and elevated moisture levels. In contrast, no clear correlation was found between thickness and water solubility, suggesting that variations in film thickness did not significantly influence solubility. Similarly, moisture content did not show a consistent relationship with solubility, indicating that higher moisture levels were not associated with either increased or decreased water solubility.

#### 3.2.3. Water Vapor Transmission Rate

The water vapor transmission rate is the ability of a film to prevent the migration of moisture from the environment to the food [20,29]. Since microbial food spoilage is often associated with high moisture permeability of food packages, a low water vapor permeability rate is important [20]. As such, a lower water vapor transmission rate indicates superior film performance in preventing moisture exchange between the food and its surrounding environment [29].

**Figure 4:**
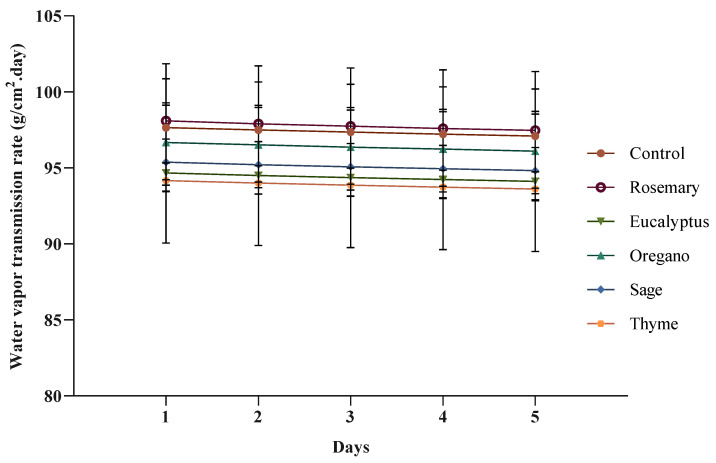
Water vapor transmission rates of the alginate and TPP film (control film) and the films incorporated with EOs.

In this work, the water vapor transmission rate of the control film and the films incorporated with EOs was determined using a gravimetric method, and the results are presented in Figure 4.

The incorporation of thyme EO into an alginate film significantly decreased the water vapor permeability rate (*p* < 0.05). On the other hand, the film incorporated with rosemary EO presented a higher water vapor permeability rate when compared to the control film, and the films with eucalyptus, oregano, and sage showed lower water vapor permeability rates; however, these differences were not significant (*p* > 0.05). The water vapor permeability rate for all films was practically constant and did not change much over time.

The water vapor transmission rate varied based on the incorporated EO. The control film and the film with rosemary EO showed the highest water vapor transmission rate values, while those with sage and thyme exhibited the lowest. There appears to be a negative correlation between film thickness and water vapor transmission rate, indicating that greater thickness may improve the barrier properties by reducing water vapor diffusion. However, no clear correlation was found between the water vapor transmission rate and either moisture content or water solubility. These results suggest that EOs affect not only the physical characteristics of the films but may also induce changes in the polymer network structure, thus influencing water vapor permeability independently of moisture retention or solubility.

In general, the incorporation of hydrophobic substances in a film, such as EOs, decreases its water vapor permeability [30]. In the present study, the films incorporated with oregano, sage, eucalyptus, and thyme EOs presented this behavior. A similar result was obtained by Motelica et al. [20] for alginate films with lemongrass EO and by Ghasemlou et al. [7] for corn-starch films incorporated with Zataria multiflora and Mentha pulegium EOs. This behavior may be attributed to the hydrophobicity of EOs and to the increased crosslinking density that prevents water mobility through the film [21].

However, some studies suggest that the water vapor permeability rate may increase in films with EOs. For instance, Han et al. [31] observed this effect in alginate and carboxymethyl cellulose films incorporated with cinnamon EO, and Jafari et al. [29] witnessed the same behavior for alginate–gelatin films with anise EO. In the present study, this was verified for the film incorporated with rosemary EO. This result may be due to the hydrophobic nature and chemical composition of EOs, which can disrupt the polymer matrix.

When EOs are incorporated into a film, they may create microstructural changes, such as pores or discontinuities within the polymer network, reducing the film’s ability to form a tight barrier and allowing more water vapor to pass through [31]. So, the results of the present work seem to indicate that the water vapor permeability rate depends on the EO incorporated into the films. Because EOs are complex mixtures of chemical compounds, their hydrophobicity is variable, which in turn influences their effectiveness as water vapor permeability reducers [32]. Additionally, the addition of lipidic substances to a film may lead to the formation of pores, changing its microstructure and thus influencing the film’s ability to act as a barrier to water [30,33].

#### 3.2.4. Water Contact Angle

Surface hydrophobicity is a crucial factor in the regulation of the sensitivity of the films to water or moisture [34], and it is typically assessed by measuring the contact angle between the film surface and a water droplet. Larger contact angles (>90°) occur on films with low wettability (hydrophobic) due to the reduced interaction between the film components and the water. Conversely, films with high wettability (hydrophilic) present smaller contact angles (<90°) because they exhibit greater interaction with water droplets [35].

**Figure 5:**
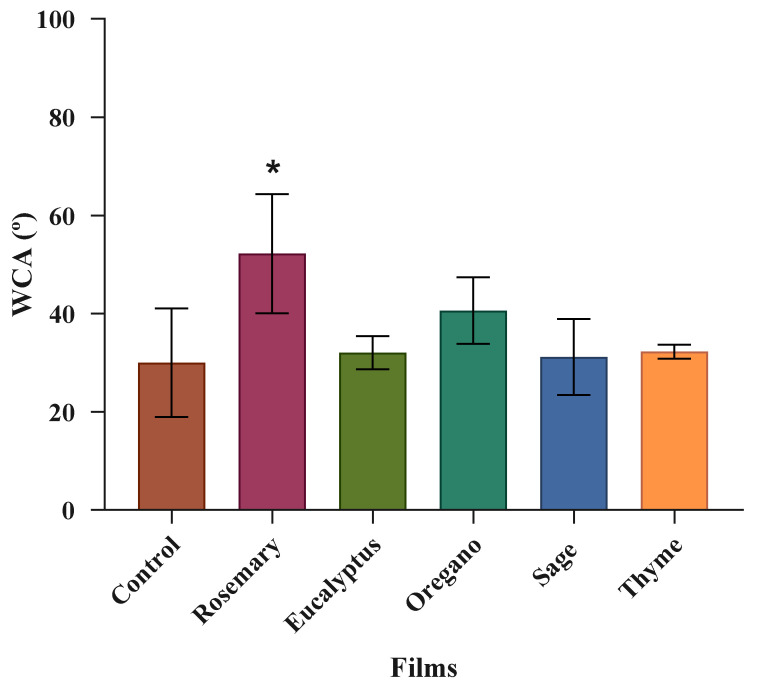
WCAs of the alginate and TPP film (control film) and the films incorporated with EOs. * mean significant differences (*p* < 0.05) between the films with rosemary oil and the control film.

In the present work, the WCAs of the films varied from 30.0° to 52.2° (Figure 5), indicating that all films have a hydrophilic behavior. This result is due to the alginate’s strong affinity for water [36].

The control film exhibited a lower WCA value. When rosemary EO was added to a film, the WCA increased significantly (*p* < 0.05) (Figure 5) compared to the control film. The incorporation of the other oils also increased the WCA value, but it was not significant (*p* > 0.05). A similar result was observed by Siracusa et al. [34] for alginate/pectin films with citral, and by Sharma et al. [37] for poly(butylene adipate-co-terephthalate)/poly(lactide) films incorporated with clove and thyme EOs. Jafari et al. [29] produced alginate/gelatin films with anise EO and obtained the same result. This author also concluded that an increase in the concentration of EO in a film increases the WCA. Therefore, adding an EO to a film, with its hydrophobic nature, raises the WCA value of a film, making it more hydrophobic [29,34,37].

WCA measurements in this study confirmed that EO incorporation influences the surface properties of alginate-based films. A negative correlation was observed between WCA and water solubility, indicating that films with more hydrophobic surfaces tend to be less soluble in water. However, no consistent correlation was found between WCA and either moisture content or film thickness. Furthermore, WCA values did not align with trends in water vapor permeability rate, suggesting that surface hydrophobicity alone does not fully determine moisture barrier behavior. These results imply that both surface characteristics and internal structural modifications induced by EOs play a role in the water resistance properties of the films.

### 3.3. Bioactivities

#### 3.3.1. Antimicrobial Activity

##### Antibacterial Activity

**Figure 6:**
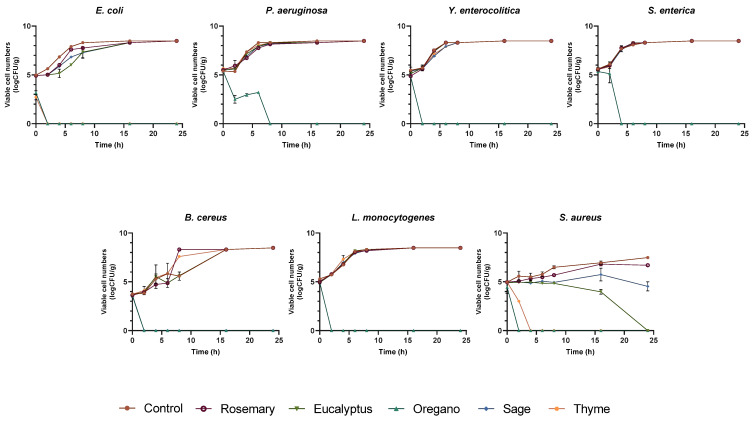
Growth inhibition curves of selected bacteria in contact with the control film and the films incorporated with the different EOs.

All films were tested against the bacterial species *S. aureus*, *B. cereus*, *L. monocytogenes*, *E. coli*, *P. aeruginosa*, *Y. enterocolitica*, and *S. enterica* serovar enteritidis using the viable cell count assay. The results are shown in Figure 6.

The film with alginate and TPP (no added EO) did not inhibit any of the studied bacteria that grew exponentially, thus acting as a positive control. This result is similar to the one reported by the authors in a previous study [15].

The film incorporated with oregano EO was the only one that could completely inhibit the growth of all studied bacteria. In fact, this film inhibited the growth of *E. coli*, *Y. enterocolitica*, *S. aureus*, *B. cereus*, and *L. monocytogenes* after two hours of contact. Still, it took four hours to completely inhibit S. enterica and eight hours to eradicate *P. aeruginosa*. The film with thyme EO inhibited *E. coli* (2 h) and *S. aureus* (4 h). The film with eucalyptus EO could only inhibit *S. aureus* after 24 h, and the film with sage EO eradicated *B. cereus* after two hours. The film incorporated with rosemary EO did not inhibit the growth of any of the bacteria under study (Figure 6).

Numerous studies have documented the incorporation of EOs into alginate films [19,22,23,29,30,33]. However, to the best of the authors’ knowledge, none of these studies have incorporated all the EOs used in this work, highlighting its novelty. Furthermore, the antibacterial activity of films is typically assessed using the disc diffusion method [2,10,30,31,38] rather than the viable cell count assay employed in this study, which simulates the real interaction between contaminant microorganisms and films more accurately. Nonetheless, it is possible to compare the results of this study with those from other research that produced films using different polymers and/or incorporated different oils. For instance, Sharma et al. [37] developed poly(butylene adipate-co-terephthalate)/poly(lactide) films incorporated with thyme and clove EOs and conducted a kinetic study against *E. coli* and *S. aureus*. They found that the film containing 10% (*v*/*v*) clove oil inhibited *E. coli* and *S. aureus*, whereas the film with thyme oil at the same concentration did not inhibit these bacteria. In the present study, however, the alginate and TPP film with thyme EO completely inhibited the growth of these bacteria at a lower oil concentration (3% (*v*/*v*)). This suggests that the film matrix may influence its antimicrobial efficacy [39], possibly due to interactions between the film components that affect the release of the EO [40].

The antibacterial activity of EOs is well known. In fact, according to Spréa et al. [14], the antibacterial activity of oregano, thyme, sage, and rosemary EOs against *S. aureus*, *B. cereus*, *L. monocytogenes*, *E. coli*, *P. aeruginosa*, *Y. enterocolitica*, and *S. enterica* present minimum inhibitory concentration values ranging from 0.1% to 2.5% (*v*/*v*). However, in the present work, when EOs were incorporated into alginate films, their antimicrobial activity decreased. Several factors may explain this phenomenon: (i) interactions between the hydroxyl groups of the oils’ phenolics and the polymeric matrix of the film [38,41], which can hinder their effectiveness, (ii) the potential loss of the highly volatile compounds of the EO during film preparation [41], and (iii) slower/controlled release of active compounds from the film, which does not occur when the crude oil is in direct contact with a microorganism [42].

##### Antifungal Activity

The antimicrobial activity of the films was also assessed against the fungal species *A. niger*, *P. expansum*, *F. verticillioides*, and *Cladosporium* sp. But, unlike bacteria, which form numerous single cells measurable as CFU/mL or CFU/g, filamentous fungi produce inseparable mycelium, making the CFU method applicable only for counting spores. Additionally, since fungi generate a larger number of spores, there is no direct correlation between fungal biomass and viable counts [43]. Consequently, the viable cell method was not employed to determine the antimicrobial activity of these organisms; instead, in this study, the antifungal activity was evaluated based on the fungi’s ability to grow on film surfaces, using a method adapted from Guimarães et al. [44]. The results were expressed as a percentage of inhibition, representing the reduction in the fungal growth on the films containing EOs compared to the growth of the fungus on the control film (Table 2).

The film incorporated with oregano EO was the one with the best antifungal activity, being able to completely inhibit the growth of all fungi tested in this study. On the contrary, the film with rosemary EO did not inhibit any of the fungal species. The thyme EO film completely inhibited the growth of *F. verticillioides* and *Cladosporium* sp. and presented an inhibition percentage (*p* < 0.05) of 36.7% for *A. niger* and 30.4% for *P. expansum*. The film with eucalyptus EO inhibited the growth of *Cladosporium* sp. completely and presented an inhibition percentage (*p* < 0.05) of 11.9% for *A. niger*; however, it did not inhibit *P. expansum* and *F. verticillioides*. The film incorporated with sage EO did not *inhibit F. verticillioides* and *Cladosporium* sp. and presented an inhibition percentage (*p* < 0.05) of 11.5% for *A. niger* and 14.5% for *P. expansum*.

Several studies have confirmed the antifungal activity of polymeric films incorporated with EOs [29,45,46,47]. However, many of these studies involved the incorporation of oils into films made from materials other than alginate [45,46,47]; to our knowledge, none have incorporated the five EOs used in this work into the films. Additionally, antifungal activity is typically assessed using the agar diffusion assay [29,45,46] rather than by calculating the inhibition percentage, as was performed in this study.

Jafari et al. [29] produced alginate/gelatin films incorporated with anise EO in a range between 0.5% and 1.5%, tested them by agar diffusion method against *A. niger*, and concluded they were able to inhibit this fungus. The authors tested films with five different EOs against the same fungus in the present study. They concluded that all films, except the one incorporated with rosemary EO, showed some growth inhibition. However, the concentration of EOs incorporated into the films, in the present study, was higher than the concentration range used by Jafari et al. [29]. Muriel-Galet et al. [48] produced ethylene vinyl alcohol films with oregano EO and tested their antifungal activity against *P. expansum*; they verified that the films completely inhibited the fungus. The present study attained a similar result for this fungal species for the film with oregano EO. Yahyaoui et al. [47] evaluated the antifungal activity against *A. niger* of polylactic acid films incorporated with rosemary, myrtle, and thyme EOs, using a similar method to the one employed in this study. They found that films with rosemary and thyme EOs exhibited higher antifungal activity against *A. niger* than those with myrtle oil. In the present research, the antifungal activity of alginate films containing rosemary and thyme EOs was tested against the same fungal species; however, the film with rosemary oil did not inhibit this microorganism, and the film with thyme EO showed an inhibition percentage of 36.7%. The antifungal properties of EOs are well established [14]. Like the antibacterial activity findings, an EO’s antifungal potential differed between the crude form and when it was incorporated into a film. This could be explained by interactions between the film polymers and active compounds of EOs, which can affect their diffusion into the medium [45].

In summary, the film containing oregano EO exhibited the most potent antimicrobial activity, effectively inhibiting all microorganisms studied in this study. The film with thyme EO also performed well, completely inhibiting *E. coli*, *S. aureus*, *F. verticillioides*, and *Cladosporium* sp. In contrast, the film with rosemary EO demonstrated the weakest antimicrobial potential, failing to inhibit any of the tested microorganisms. The eucalyptus and sage EOs films showed some inhibitory effects against certain bacteria and fungi.

A negative correlation appears to exist between film thickness and antimicrobial activity. Thinner films (e.g., oregano: 0.12 mm; thyme: 0.16 mm) exhibited stronger antimicrobial effects, whereas thicker films (e.g., sage: 0.29 mm; eucalyptus: 0.24 mm) were less effective or demonstrated delayed antimicrobial action. This trend may be attributed to the fact that thinner films likely facilitate a more efficient release and diffusion of essential oils, thereby enhancing their antimicrobial efficacy.

Given their antimicrobial potential, these films show promise for preserving fresh foods, such as fruits and vegetables, as they can inhibit certain spoilage and pathogenic microorganisms commonly associated with these products.

#### 3.3.2. Antioxidant Activity

**Figure 7:**
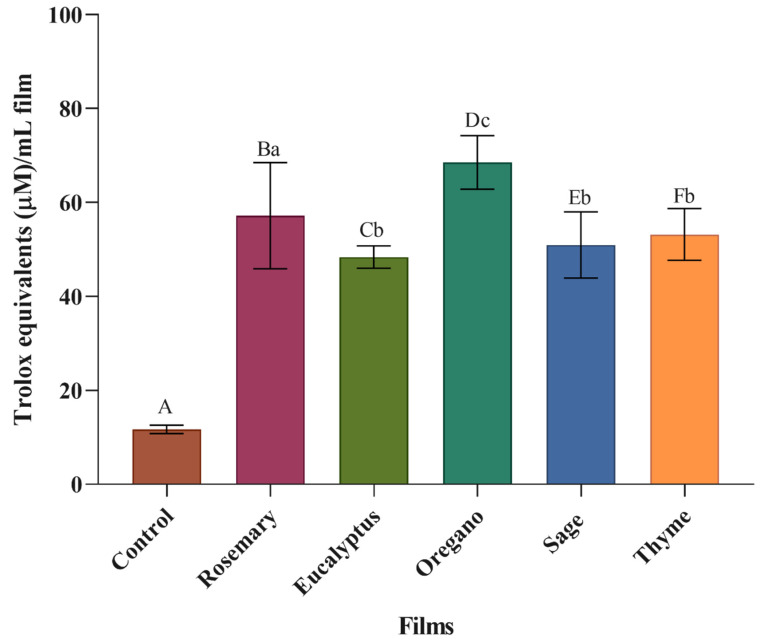
Antioxidant activity of the control film and the films incorporated with the different EOs. Upper-case letters mean significant differences (*p* < 0.05) between the films with EOs and the control film, and lower-case letters mean significant differences (*p* < 0.05) between the films with different EOs.

The antioxidant potential of the films was assessed by kinetically measuring the scavenging of the cationic radical ABTS+·. Initially, the films dissolved in water were tested, but the antioxidant activity values were nearly negligible and likelynot representative. Subsequently, the antioxidant activity was evaluated directly on the solid films, as performed in a previous study [15]. However, due to the high volatility of EOs, the results were inconsistent. Therefore, a kinetic test was selected to better understand the release of EOs from the films and to simulate their interaction with food surfaces more accurately. The results are shown in Figure 7.

The control film (11.65 µM Trolox equivalents/mL of film) exhibited minimal antioxidant activity compared to the films containing EOs. However, the antioxidant activity of the control film was not zero, as alginate itself possesses inherent antioxidant properties [49].

EOs are rich in phenolic compounds, which give them strong antioxidant activity. The active groups in EOs interrupt the free radical chain reaction by acting as hydrogen donors that neutralize the radicals [50]. As a result, the incorporation of EOs into the alginate films, as expected, significantly (*p* < 0.05) enhanced their antioxidant activity. The film incorporated with oregano EO presented higher (*p* < 0.05) antioxidant activity (68.51 µM Trolox equivalents/mL of film). In contrast, eucalyptus EO film had the lowest (*p* < 0.05) antioxidant potential (48.35 µM Trolox equivalents/mL of film).

Similar to the antimicrobial activity, a negative correlation also appears to exist between film thickness and antioxidant activity. Thinner films (e.g., oregano: 0.12 mm) demonstrated stronger antioxidant effects, while thicker films (e.g., eucalyptus: 0.24 mm) were less effective. This may be due to the fact that thinner films enable a more efficient release and diffusion of essential oils, thereby enhancing the interaction of antioxidant compounds with free radicals.

Owing to their antioxidant properties, these films show promise for preserving fresh foods such as fruits and vegetables, as they help maintain nutritional quality and protect bioactive compounds, thereby slowing down product deterioration.

Numerous studies have shown that incorporating EOs into alginate films [18,22] and films made from other polymers [51,52] enhances their antioxidant potential. However, in those studies, the antioxidant activity is typically measured in the film-forming solution [51,53], rather than the film itself, which does not accurately reflect the conditions when the film is applied to a food surface. Few studies determine the antioxidant activity of films by kinetically measuring the scavenging of the cationic radical ABTS+·. DiCastillo et al. [54] used a similar method on methyl-cellulose films with murta fruit extract and found that the extract enhanced the films’ antioxidant activity. However, their study did not focus on incorporating EOs into films. To the authors’ knowledge, no previous research has employed the kinetic ABTS scavenging method to evaluate the antioxidant activity of alginate films containing EOs, highlighting the novelty of this work.

## 4. Conclusions

EOs are natural substances recognized for their bioactive potential, making them promising alternatives to conventional food preservatives. However, their strong aroma/flavor and high volatility present challenges. Incorporating EOs into edible films can help overcome these limitations by controlling their release and reducing their sensory impact. This study focused on producing and characterizing edible alginate-based films incorporated with rosemary, eucalyptus, oregano, sage, and thyme EOs.

The films were thoroughly characterized, with particular attention to their antimicrobial and antioxidant properties. The incorporation of EOs influenced several physical attributes, including thickness, moisture content, and water solubility. The water vapor transmission rate also varied depending on the specific EO used. WCA measurements confirmed that EO incorporation alters the surface properties of the alginate-based films. The films, particularly those with oregano EO, demonstrated strong antimicrobial and antioxidant activities.

Moreover, since the films were made from natural materials, they have the potential to serve as eco-friendly alternatives to conventional synthetic plastics, which contribute to environmental pollution.

Nevertheless, further studies are necessary to expand the mechanical characterization of these films, including tests for tensile strength, elongation at break, and puncture resistance. The evaluation of additional barrier properties, such as oxygen and carbon dioxide permeability, is also important. Thermal analyses, including TGA and DSC, along with surface characterization techniques such as SEM and AFM, would provide valuable insights into the films’ structural and morphological features. Moreover, assessing the cytotoxicity and genotoxicity of these materials is crucial to ensure their safety. Finally, evaluating their bioactivity in real food matrices is essential to confirm their effectiveness in practical applications.

## Figures and Tables

**Table 1:** Values of thickness, moisture, and solubility in water for the control film and the films incorporated with EOs.

	Thickness (mm)	Moisture (%, Dry Basis)	* Solubility in Water (%)
**Control**	0.17 ± 0.01 ^A^	29.54 ± 0.03 ^A^	95.10 ± 2.38 ^A,a^
**Rosemary**	0.17 ± 0.01 ^A,a^	32.54 ± 0.38 ^A,a^	87.13 ± 4.54 ^A,a^
**Eucalyptus**	0.24 ± 0.02 ^B,b,e^	35.13 ± 1.27 ^B,a^	89.17 ± 13.85 ^A,a^
**Oregano**	0.12 ± 0.01 ^B,c,f^	35.24 ± 1.05 ^C,a,d^	83.72 ± 0.04 ^A,a^
**Sage**	0.29 ± 0.03 ^B,d^	37.79 ± 1.05 ^D,b,d^	94.29 ± 3.98 ^A,a^
**Thyme**	0.16 ± 0.01 ^A,a^	37.11 ± 0.39 ^E,c,d^	86.65 ± 1.52 ^A,a^

Upper-case letters in the same column mean significant differences (*p* < 0.05) between the films with EOs and the control film, and lower-case letters in the same column mean significant differences (*p* < 0.05) between the films with different EOs. * Statistical analysis using a non-parametric test followed by the Kruskal–Wallis test.

**Table 2:** Antifungal activity of the films incorporated with EOs against some fungal species.

Inhibition (%)					
Fungus	Rosemary	Eucalyptus	Oregano	Sage	Thyme
***A. niger***	NI	11.9 ± 2.2 ^a^	100 ± 0 ^b^	11.5 ± 7.7 ^c^	36.7 ± 1.6 ^d^
***P. expansum***	NI	NI	100 ± 0 ^a^	14.5 ± 9.1 ^b^	30.4 ± 6.0 ^c^
***F. verticillioides***	NI	NI	100 ± 0 ^a^	NI	100 ± 0 ^a^
***Cladosporium* sp.**	NI	100 ± 0 ^a^	100 ± 0 ^a^	NI	100 ± 0 ^a^

NI: No inhibition. Different letters in the same row mean statistically significant differences (*p* < 0.05 according to Tukey’s HSD test).

## Data Availability

The original contributions presented in this study are included in the article. Further inquiries can be directed to the corresponding author.
